# Rapid versus traditional qualitative analysis using the Consolidated Framework for Implementation Research (CFIR)

**DOI:** 10.1186/s13012-021-01111-5

**Published:** 2021-07-02

**Authors:** Andrea L. Nevedal, Caitlin M. Reardon, Marilla A. Opra Widerquist, George L. Jackson, Sarah L. Cutrona, Brandolyn S. White, Laura J. Damschroder

**Affiliations:** 1 Center for Innovation to Implementation (Ci2i), VA Palo Alto Health Care System (152-MPD), 795 Willow Road, Building 324, Menlo Park, CA 94025 USA; 2 Veterans Affairs (VA) Center for Clinical Management Research, Ann Arbor Healthcare System, 2215 Fuller Rd. (152), Ann Arbor, MI 48105 USA; 3 Center of Innovation to Accelerate Discovery and Practice Transformation (ADAPT), Durham VA Health Care System, Durham, USA; 4 Department of Population Health Science, Duke University, Durham, USA; 5 Division of General Internal Medicine, Duke University, Durham, USA; 6 Department of Family Medicine and Community Health, Duke University, Durham, USA; 7 Center for Healthcare Organization & Implementation Research, Bedford & Boston VA Medical Centers, Boston, USA; 8 Department of Population and Quantitative Health Sciences, University of Massachusetts Medical School, Worcester, USA; 9 Division of General Internal Medicine, University of Massachusetts Medical School, Worcester, USA

**Keywords:** Consolidated Framework for Implementation Research (CFIR), Qualitative methods, Rapid analysis, Implementation science, Veterans

## Abstract

**Background:**

Qualitative approaches, alone or in mixed methods, are prominent within implementation science. However, traditional qualitative approaches are resource intensive, which has led to the development of rapid qualitative approaches. Published rapid approaches are often inductive in nature and rely on transcripts of interviews. We describe a deductive rapid analysis approach using the Consolidated Framework for Implementation Research (CFIR) that uses notes and audio recordings. This paper compares our rapid versus traditional deductive CFIR approach.

**Methods:**

Semi-structured interviews were conducted for two cohorts of the Veterans Health Administration (VHA) Diffusion of Excellence (DoE). The CFIR guided data collection and analysis. In cohort A, we used our traditional CFIR-based deductive analysis approach (directed content analysis), where two analysts completed independent in-depth manual coding of interview transcripts using qualitative software. In cohort B, we used our new rapid CFIR-based deductive analysis approach (directed content analysis), where the primary analyst wrote detailed notes during interviews and immediately “coded” notes into a MS Excel CFIR construct by facility matrix; a secondary analyst then listened to audio recordings and edited the matrix. We tracked time for our traditional and rapid deductive CFIR approaches using a spreadsheet and captured transcription costs from invoices. We retrospectively compared our approaches in terms of effectiveness and rigor.

**Results:**

Cohorts A and B were similar in terms of the amount of data collected. However, our rapid deductive CFIR approach required 409.5 analyst hours compared to 683 h during the traditional deductive CFIR approach. The rapid deductive approach eliminated $7250 in transcription costs. The facility-level analysis phase provided the greatest savings: 14 h/facility for the traditional analysis versus 3.92 h/facility for the rapid analysis. Data interpretation required the same number of hours for both approaches.

**Conclusion:**

Our rapid deductive CFIR approach was less time intensive and eliminated transcription costs, yet effective in meeting evaluation objectives and establishing rigor. Researchers should consider the following when employing our approach: (1) team expertise in the CFIR and qualitative methods, (2) level of detail needed to meet project aims, (3) mode of data to analyze, and (4) advantages and disadvantages of using the CFIR.

**Supplementary Information:**

The online version contains supplementary material available at 10.1186/s13012-021-01111-5.

Contributions to the literature

- Published rapid qualitative analysis approaches often use transcripts; our approach shows how notes and verification with audio recordings can be used to ensure rigor while saving time and eliminating transcription costs.
- Published rapid qualitative analysis approaches often utilize inductive approaches; our approach shows how to conduct deductive rapid analysis using the Consolidated Framework for Implementation Research (CFIR), which allows researchers to compare results more easily across studies.
- CFIR users have expressed difficulty using the framework because our traditional analysis approach is resource intensive; the rapid analysis approach described here may facilitate the use of the CFIR for experienced users.

## Background

Qualitative methods are invaluable for gathering in-depth information about “how and why efforts” to implement Evidence-Based Innovations (EBIs) succeed or fail [1]. As a result, qualitative approaches (alone or within mixed methods) are foundational for implementation scientists seeking to identify and understand factors that help or hinder the implementation and use of EBIs in real-world settings [2, 3]. Traditional qualitative approaches, however, are resource intensive, which challenges constrained study timelines and budgets. This is especially problematic in studies where scientists need real-time data to inform the process of implementation [4].

Consequently, qualitative researchers are working to develop methods that balance rigor and efficiency. The need for this balance is particularly salient in healthcare, where treatments and interventions are rapidly evolving, and evaluations of such interventions are constrained by limited timelines, funding, and staffing [5]. As a result, rapid assessment, which often involves streamlined processes for qualitative data collection and analysis, is gaining increased attention as a way to support quicker implementation and dissemination of EBIs to reduce delays in translating clinical research into practice [6–10].

An important element of rapid assessment is rapid qualitative analysis, which is the focus of this paper. Traditionally, qualitative analysis approaches have been resource intensive and occur over a longer timeframe; they include, but are not limited to, constant comparison, content, discourse, or thematic analysis [11–16]. Many traditional qualitative analysis approaches include in-depth manual coding of transcripts using software programs. In contrast, rapid qualitative analysis is deliberately streamlined and designed to be less resource intensive in order to meet a shorter timeframe [17–19]. Rapid qualitative analysis may involve eliminating transcription altogether or speeding up transcription processes [19] and then summarizing data into post-interview notes, templates based on the interview guide, and/or matrix summaries rather than in-depth manual coding of transcripts [9, 17–21]. Though rapid analysis is not wedded to a particular approach (e.g., content or thematic), some traditional qualitative analysis approaches may be more difficult to streamline. Rapid qualitative analysis is crucial when results are needed to quickly develop or modify implementation strategies and/or inform stakeholders or operational partners [5, 7, 9, 10, 19]. Rapid qualitative analysis is also useful during longitudinal implementation research since data points can become unwieldy, and results may be needed to inform future waves of data collection [22].

Hamilton developed a rapid qualitative analysis approach that summarizes transcript data into templates using domains aligned with interview questions; summary points are then distilled into a matrix organized by domain and participant for analysis and interpretation [18]. Gale et al. adapted this rapid approach in a process evaluation of academic detailing and compared it with a traditional analysis approach [23]. Their rapid approach involved summarizing transcripts into a template and then mapping themes onto the Consolidated Framework for Implementation Research (CFIR), a determinant framework that defines constructs across five domains of potential influences on implementation [23–26]. Gale et al. demonstrated consistency between results from rapid qualitative analysis versus traditional qualitative analysis. The traditional approach, however, “took considerably (69 days) longer than the rapid analysis to complete” [23]. Similarly, Holdsworth et al. noted that their modified version of rapid qualitative analysis “produced contextually-rich information” and can be used to save “days and weeks of costly transcription and analysis time” [27]. Except for Taylor et al.’s [20] comparison of rapid and thematic analysis, most rapid analysis literature focuses on daily duration and does not quantify reductions in analyst hours and costs at the activity level versus the project overall.

The rapid approaches described by Hamilton and Gale et al. rely on verbatim transcripts, which means teams must wait for transcription to be completed to proceed with rapid or traditional analyses. In contrast, Neal et al. [28] developed an approach to rapidly identify themes directly from audio recordings. However, Gale et al. [23] noted that because this approach relies on general domains, rather than framework informed codes, it “limits one’s ability to compare findings across projects unless findings are [subsequently] mapped to a framework.” As implementation scientists using the CFIR to guide our evaluations, we sought to build on prior rapid analysis approaches by developing a CFIR informed deductive rapid analysis process using notes and audio recordings. The objective of this article is to compare two different qualitative analysis processes using the CFIR: a traditional deductive approach using transcripts and a rapid deductive approach using notes and audio recordings.

## Methods

### Evaluation background

We conducted a mixed-methods evaluation of the Veterans Health Administration (VHA) Diffusion of Excellence (DoE), which seeks to identify and diffuse EBIs. These EBIs include innovations supported by evidence from research studies and administrative or clinical experience [29, 30] and strive to address patient, staff, and/or facility needs. The DoE hosts an annual “Shark Tank” competition, in which VHA leaders compete to implement an EBI with 6 months of external implementation support; for additional detail, see previous publications [31–34]. As part of a national evaluation of the DoE, we identified barriers and facilitators to the implementation of these EBIs in VHA facilities using semi-structured interviews [31]. The qualitative interview and analysis team included CR (MPH, a senior qualitative analyst and CFIR expert user) and AN (PhD, a senior qualitative methodologist and CFIR intermediate user). Per regulations outlined in VHA Program Guide 1200.21, this evaluation has been designated a non-research quality improvement activity.

### Methods for the traditional and rapid approaches

#### Data collection: semi-structured interviews

Data collection methods were the same across both approaches; in effect, they will not be discussed in detail in this paper. In brief, we conducted semi-structured telephone interviews with DoE participants involved with implementing an EBI; for additional detail, see previous publications [31, 35]. Interview guides were informed by the CFIR (see Additional File 1). Cohort A included 57 interviews across 17 facilities (1–4 interviews/facility) from June 2017 to September 2017; because one facility only had one interview, the need to aggregate data for that facility was eliminated. Cohort B included 72 interviews across 16 facilities (3–6 interviews/facility) from May 2019 to September 2019. Although cohort B included more interviews, the interviews were on average shorter (approximately 30 min), so both cohorts had approximately 50 audio hours total.

##### Data analysis: traditional and rapid approaches

The steps in our CFIR-based deductive traditional and deductive rapid qualitative analysis approaches are described in Table 1. The traditional CFIR approach is described in detail on www.cfirguide.org and in several publications [31, 36–38]. Our traditional CFIR approach is a form of directed content analysis [11] using transcripts and consisted of the following steps:

1. The analysts independently coded verbatim transcripts using Dedoose [39], a collaborative qualitative software program. The codebook included deductive CFIR constructs as well as inductive codes not captured in the CFIR that were relevant to the evaluation. Analysts used comments within coding software to flag sections of text for discussion or add additional notes.
2. The analysts met weekly to adjudicate differences in coding.
3. The primary analyst exported and aggregated coded data in *MS Word CFIR facility memos* (one for each facility). See Table 2 and Additional File 2.
4. The primary analyst summarized and rated coded data and wrote high-level facility summaries in each facility memo. The secondary analyst reviewed the primary analyst’s drafts of the facility memos and edited the summaries, ratings, and high-level facility summaries. Ratings were based on two factors: (1) valence (positive or negative influence on implementation) and (2) strength (weak or strong influence on implementation). Analysts used comments and highlighting in the facility memo to flag sections of text for discussion. Completed facility memos ranged from 68 to 148 pages with an average of 108 pages.
5. The analysts met weekly to adjudicate differences and refine the codebook.
6. The primary analyst copied the summaries, ratings, and high-level facility summaries from each facility memo into the *MS Excel CFIR construct by facility matrix* for interpretation; the matrix included all codes from the codebook (both deductive and inductive codes) as well as a row for high-level facility summaries. See Table 3 and Additional File 3.

**Table 1:** Traditional versus rapid approach using the CFIR

	**Traditional Deductive CFIR Approach (Cohort A)**	**Rapid Deductive CFIR Approach (Cohort B)**
	**Data Management**	
	Create MS Word CFIR Facility Memo Template. Create project and codebook in qualitative software program. See Table 2 and Additional File 2.	N/A
	^*a*^*Create MS Excel CFIR Construct by Facility Matrix Template (CFIR constructs as rows and facilities as columns). See Additional File 3.*	
**Time**	**1 h/project set-up**	**.5 h/project set-up**
	^b^Transcribe audio recordings.	N/A
	De-identify and import transcripts into software program.	N/A
**Time**	**.5 h/interview**	**0 h/interview**
	Copy and paste summaries, ratings, and rating rationales into matrix. See Table 3 and Additional File 3.	N/A
**Time**	**.5 h/facility**	**0 h/facility**
**Total Time**	**1 h/project set-up + (.5 h/interview + .5 h/facility)**	**.5 h/project set-up**
	**Data Collection**	
	^*a*^*Conduct and record semi-structured interviews. See Additional File 1.*	
**Total Time**	**1 h/interview**	**1 h/interview**
	**Data Analysis: Coding and Adjudication Process: Process is repeated for each interview**	
	Primary analyst: Code verbatim transcript independently in qualitative software program and use comments as needed.	^c^Primary analyst: Write notes during interview and “code” into matrix immediately after interview; use comments and highlight areas that need clarification or timestamps. Write (and update) facility summary with each interview. See Table 3 & Additional File 3.
**Time**	**1.5 h/interview**	**1.72 h/interview**
	Secondary analyst: Code verbatim transcript independently and use comments as needed.	Secondary analyst: Review notes in matrix, listen to audio recording, and use comments and different colored text to highlight additional notes, edits, quotes, or timestamps.
	**Traditional Qualitative Approach (Cohort A)**	**Rapid Qualitative Approach (Cohort B)**
**Time**	**2.5 h/interview**	**1.70 h/interview**
	Primary analyst: Review coding for differences and meet with secondary analyst to reach consensus.	Primary analyst: Review notes for differences and meet with secondary analyst to reach consensus.
**Time**	**1.5 h/interview**	**.5 h/interview**
**Total Time**	**5.5 h/interview**	**3.92 h/interview**
	**Data Analysis: Rating and Adjudication Process: Process is completed for each facility**	
	Export coded data and aggregate in facility memo; memos were an average of 108 pages/facility. *See Table 2 and Additional File 2.*	N/A
	Primary Analyst: Review all data (all participants in facility) in facility memo and write summary for each CFIR construct and the facility overall. *See Table 3.*	Primary Analyst: Review all notes (all participants in facility) in facility column in matrix (see above); data is already in note form and facility summary has been written. *See Table 3 and Additional File 3.*
	Primary Analyst: Rate each CFIR construct in facility memo and provide rating rationale.	Primary Analyst: Rate each CFIR construct in facility column in matrix and provide rating rationale.
**Time**	**8 h/facility**	**1.69 h/facility**
	Secondary Analyst: Review facility memo and edit summaries, ratings, and rating rationales.	Secondary Analyst: Review facility column in matrix and edit ratings and rating rationales
**Time**	**4 h/facility**	**1.23 h/facility**
	Primary analyst: Review facility memo for differences and meet with secondary analyst to reach consensus.	Primary analyst: Review facility column in matrix for differences and meet with secondary analyst to reach consensus
**Time**	**2 h/facility**	**1 h/facility**
**Total Time**	**14 h/facility**	**3.92 h/facility**
	**Data Interpretation:**	
	^*a*^*Review and interpret data by facility; write facility level summaries.*	
	^*a*^*Review and interpret data by construct; organize facilities by implementation outcomes and identify constructs that manifested positively across facilities, negatively across facilities, or distinguished between facilities with high and low implementation success.*	
**Total Time**	**100 h/project**	**100 h/project**

^a^These aspects are the same for both the traditional and rapid deductive CFIR approaches

^b^In this project, the team paid for transcription. This resulted in a transcription cost difference and an approximate 2 – 6-week delay while waiting for transcription to be completed, but not an increase in analyst time on the project

^c^If the primary analyst is unable to take notes during the interview and/or code them immediately after the interview, they could listen to the audio following the interview. Though this would add additional time to the analysis process, it may provide an alternative for teams conducting back-to-back interviews in the same day (e.g., during site visits)

**Table 2:** Abridged CFIR facility memo template

Analysts:	
Facility:	
Interview participants:	
**High-level facility summary:**	
[Provide high-level summary of the facility]	
**I. Innovation characteristics**	
**A. Innovation source**	
*RATING: OVERALL __ (ANALYST 1 __, ANALYST 2 __)*	
*Summary:*	
[Provide summary of data.]	
*Rationale:*	
[Provide a rationale for rating.]	
*Data:*	
[Copy coded data from software.]	
**B. Evidence, strength, and quality**	
*RATING: OVERALL __ (ANALYST 1 __, ANALYST 2 __)*	
*Summary:*	
[Provide summary of data.]	
*Rationale:*	
[Provide a rationale for rating.]	
*Data:*	
[Copy coded data from software.]	

This is an abridged version of the CFIR facility memo template; the unabridged memo contains all CFIR domains and constructs. See Additional File 2

**Table 3:** Snippet of CFIR construct by facility matrix

Approach	Traditional approach (cohort A)	Rapid approach (cohort B)
**Inner setting**		
**Leadership engagement (LE)**	^a^Overall rating −2 **Summary:** The implementation leader tried to brief the [Leadership Role 1] when she returned from the DoE Base Camp, but “she was very busy that week, so I was told to maybe meet with the [Mid-Level Leadership Role 1] instead.” The [Key Stakeholder 1] believes one of the biggest barriers to implementation was unstable and acting leadership; most of the leadership team was acting or missing during implementation, which has required them to brief and re-brief new leadership. **Rationale:** Leadership was minimally engaged throughout implementation, which [Key Stakeholder 1] felt was a big barrier to implementation, warranting a −2 rating.	Overall rating +2 **Summary:** ^b^P1: Leadership was very engaged. P2: The [P2] was responsible for “dislodging” barriers up the chain as necessary, e.g., reaching out to leadership to support training. He states that site leadership “mandated” or “deeply inspired” them to set time aside to be trained. P3: She felt leadership was very engaged based on (1) [Leadership Role 1] bidding; (2) [Leadership Role 2] encouraging staff to participate with [EBI Name] Day; (3) [Leadership Role 3] adding it to the pay-for-performance plan. **Rationale:** Leadership provided ongoing tangible support and incentives, warranting a +2 rating.
**Available resources (AR)**	Overall rating: X **Summary:** Time was limited both for implementation and administration of the practice; it was a collateral duty for the implementation leader and given that [department] was short-staffed, [Role 1] had limited time to complete assessments. However, they did have funding to buy [equipment]; the [Key Stakeholder 1] was able to give them money from another VA program. **Rationale:** Important resources were both available (funding) and unavailable (dedicated time), warranting an X rating.	Overall rating +1 **Summary:** P1: It was hard for the implementation leaders to have time “carved out”; if there was one “pearl” from her, it is that bids should include time. She should not have to advocate for them to have time. Even if they were ultimately supported, she knows the implementation leader experienced frustration related to lack of time in the beginning. P2: Site had equipment already in place. **Rationale:** Although the implementation leader did not initially have dedicated time, important resources were ultimately available to support implementation (equipment, dedicated time), warranting a + 1 rating.

^a^Ratings were determined based on two factors: (1) valence (positive or negative influence on implementation) and (2) strength (weak or strong influence on implementation). Ratings ranged from +2 to −2, including neutral (0), mixed (X), and missing (M)

^b^The matrix in the rapid approach included the role of participants because the primary analyst entered notes into the matrix after each interview

In contrast, our rapid CFIR approach is a form of directed content analysis [11] using interview notes and verification with audio recordings, which consisted of the following steps:

1. The primary analyst took notes and captured quotations during interviews. Immediately after the interviews, the primary analyst “coded” the notes into the *MS Excel CFIR construct by facility matrix* and noted when additional detail or a timestamp was needed. The secondary analyst then reviewed the matrix, listened to the audio recordings, and edited and built upon the primary analyst’s notes. Analysts coded based on a codebook with deductive CFIR constructs as well as inductive codes not captured in the CFIR that were relevant to the evaluation. Analysts used comments and highlighting in the matrix to flag sections of text for discussion.
2. Analysts met weekly to adjudicate differences and refine the codebook.
3. The primary analyst reviewed notes, rated CFIR constructs, and wrote a high-level facility summary for each facility in the matrix; the secondary analyst reviewed the matrix and edited ratings and high-level facility summaries. Ratings were determined based on two factors: (1) valence (positive or negative influence on implementation) and (2) strength (weak or strong influence on implementation). See Table 3 and Additional File 3.
4. Analysts met weekly to adjudicate differences.

##### Data interpretation: facility and construct analyses

Data interpretation methods were the same across both approaches and are discussed in detail on www.cfirguide.org. In brief, the analysts completed the following steps: (1) facility (case) analyses, to identify constructs that influenced implementation outcomes in each facility, and (2) construct analyses, to identify CFIR constructs that manifested positively or negatively across facilities or distinguished between facilities with high and low implementation success.

### Methods for comparing traditional and rapid approaches

#### Comparing time and transcription costs

The team tracked time for data management, data collection, data analysis, and data interpretation for both approaches using MS Excel spreadsheets. Staff time for these tasks is based on hours. We also combined both analyst’s funded effort to determine the total available analyst hours for our evaluation. Transcription costs were obtained from invoices from a centralized VHA qualitative interview transcription service.

#### Comparing effectiveness and rigor

The team did not plan to compare the effectiveness or rigor of our traditional versus rapid approach (see the “Limitations” section). As a result, we defined and assessed these aspects retrospectively. Effectiveness was measured by whether we met our evaluation objective in each approach. Rigor was measured primarily by assessing the credibility of each approach, i.e., if evaluation processes established confidence that the results were accurate [40, 41].

## Results

### Comparing traditional and rapid approaches

#### Time and transcription costs

**Fig. 1:**
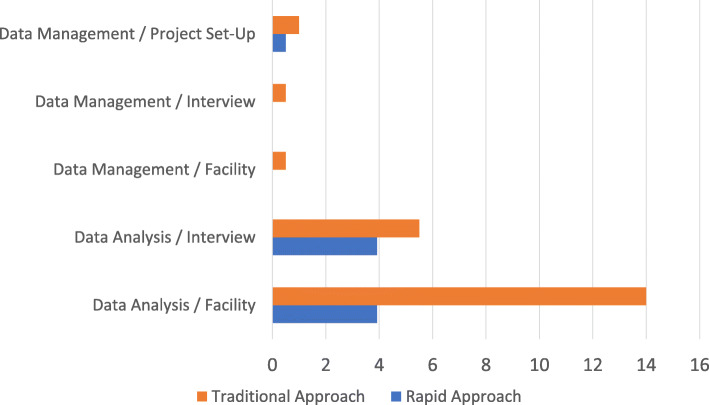
Comparison of analysis hours for the Traditional CFIR Approach (Cohort A) versus the Rapid CFIR Approach (Cohort B). This graph does not include data collection or data interpretation because both were equal across Cohort A and B

The traditional approach required more time than the rapid approach and included transcription costs. Cohort A, using the traditional deductive CFIR approach, required 683 total hours and $7250 in transcription costs. Cohort B, using the rapid deductive CFIR approach, required 409.5 total hours with no transcription costs. In effect, the rapid approach required 273.5 fewer total hours and saved $7250 in transcription costs. The evaluation funded two analysts with a combined total of 1305 h available for each year. Cohort A required 52.3% (683/1305 h) of the available hours while cohort B required 31.4% (409.5/1305 h) of the available hours, representing a significant reduction in time within the broader context of the evaluation. However, time savings during rapid analysis varied by phase, with the largest savings during the facility-level analysis. The following sections provide a summary of analyst hours and transcription costs for both approaches. See Table 1, Table 4, and Fig. 1 for additional description.

**Table 4:** Traditional versus rapid approach: differences in analyst hours and transcription costs

**Hours**	**Traditional CFIR approach (cohort A)**	**Rapid CFIR approach (cohort B)**	**Differences in hours**
^a^Total data	**50 interview audio hours across 16 facilities**		**0 h**
Data management	**34 total hours** 1 h/project set-up = 1 .5 h × 50 interviews = 25 .5 h × 16 facilities = 8	**.5 total hours** .5 h/project set-up = .5 0 h × 50 interviews = 0 0 h × 16 facilities = 0	**33.5 h**
Data collection	**50 total hours**	**50 total hours**	**0 h**
Data analysis: interviews	**275 total hours** 5.5 h × 50 interviews	**196 total hours** 3.92 h × 50 interviews	**79 h**
Data analysis: facilities	**224 total hours** 14 h × 16 facilities	**63 total hours** 3.92 h × 16 facilities	**161 h**
Data interpretation	**100 total hours**	**100 total hours**	**0 h**
**Total hours**	**683 h**	**409.5 h**	**273.5 h**
**Transcription cost**	**Traditional CFIR approach (cohort A)**	**Rapid CFIR approach (cohort B)**	**Differences in cost**
Transcription	**$7250** $145/h × 50 h	**$0**	**$7250**

^a^Cohort A included 57 interviews across 17 facilities (1–4 interviews/facility); because one facility only had one interview, the need to aggregate data for that facility was eliminated. In effect, these calculations use 16 facilities for both cohorts. Cohort B included 72 interviews across 16 facilities (3–6 interviews/facility). However, due to a higher proportion of 30-min interviews for cohort B, both cohorts had approximately 50 audio hours

#### Data management

Data management in the traditional approach required 1 h to set-up the project and .5 h/interview plus .5 h/facility. In contrast, data management in the rapid approach required only .5 h to set-up the project with no other time needed. As shown in Table 1, the rapid approach eliminated data management steps except for creating the MS Excel CFIR construct by facility template. As a result, the rapid approach reduced analys time by 33.5 h. Though not directly impacting analyst hours, transcripts were not received for 2–6 weeks following interviews, significantly delaying analysis for the traditional approach. See Table 1, Table 4, and Fig. 1.

#### Data collection: semi-structured interviews

Data collection methods were the same across both approaches and the total number of audio hours was roughly equivalent between cohorts A and B; in effect, there were no significant differences in analyst hours between approaches. However, the rapid approach required blocking approximately 3 h for each interview: approximately 1 h for the interview plus 1–2 h to process the notes and “code” them into the CFIR construct by facility matrix immediately following the interview. The analyst’s immediate recall of the interview helped bolster the accuracy of the notes but intensified effort and cognitive load on interview days.

#### Data analysis

Data analysis in the traditional approach required 5.5 h/interview plus 14 h/facility versus 3.92 h/interview plus 3.92 h/facility in the rapid approach. In effect, the rapid approach reduced analys time by 79 h (275 versus 196 for traditional and rapid, respectively). The largest contributor to this reduction in analyst hours was in the facility-level analysis phase; where the rapid approach required 63 h, the traditional approach required 224 h. This difference was a result of how and when data were condensed and aggregated. In the traditional deductive CFIR approach, all coded data were aggregated in facility memos that were approximately 108 pages long; due to the relationships that often exist between constructs, the memos often included the same segments of text under multiple constructs. As a result, the same pieces of data were reviewed multiple times in full by each analyst independently before the data were condensed in the matrix. In contrast, the rapid deductive CFIR approach condensed data prior to aggregating by facility and was completed first by the primary analyst. Relationships between constructs were described once in the matrix, and notes in other cells referred back to this description, thus eliminating multiple references to the same data. The secondary analyst then built upon and confirmed the data in the matrix by listening to the audio recording. See Table 1, Table 4, and Fig. 1.

#### Data interpretation

Data interpretation methods were the same across both approaches, which consisted of reviewing the CFIR construct by facility matrix. Both approaches took approximately 100 h for data interpretation. See Table 1, Table 4, and Fig. 1.

#### Effectiveness and rigor

There were substantial differences in the number of hours and transcription costs between the traditional and rapid approaches; however, both approaches were systematic and there was concordance among many of the evaluation phases. Even when the analysis steps were different, both approaches followed the same general approach from data collection through data interpretation (see Table 1). Although data werecondensed earlier in the rapid approach than the traditional approach, i.e., following the interview versus following the facility memo, the depth of the data in the final matrices was similar for both approaches. For example, both matrices included brief direct quotes from participants. As a result, both approaches were effective in meeting our overall goal for the evaluation; we were able to identify and describe the factors influencing implementation in a high level of detail. However, the rapid approach also allowed us to share formal results more quickly with our operational partners (see Table 5).

**Table 5:** Traditional deductive CFIR approach versus rapid deductive CFIR approach: effectiveness and rigor

Domain	Traditional CFIR approach	Rapid CFIR approach
**Effectiveness: evaluation objectives**		
Ability to identify and describe implementation determinants	Yes	Yes
Ability to provide rapid feedback to operational partners	No (preliminary results only)	Yes
**Rigor: evaluation processes**		
**Credibility**		
Analyst authority: We had analysts with expertise in both qualitative methods and the CFIR	Yes	Yes
Data accuracy: We used two analysts/interview and maintained access to the raw data in order to verify the accuracy of data, especially quotations	Yes (transcripts and audio recordings)	Yes (audio recordings)
Data organization: We used matrices, allowing us to parse out and synthesize data as needed	Yes	Yes
**Dependability**		
Data comparability: We used the same interviewers and semi-structured interview guide (based on the CFIR) to ensure data was comparable across participants and facilities	Yes	Yes
Coding comparability: We used the same analysts and framework to ensure coding was comparable across participants and facilities	Yes	Yes
Analysis audit trail: We documented keys phases of analysis and edits in memos and/or matrices	Yes	Yes
**Confirmability**		
Data triangulation: We interviewed multiple participants at each site, allowing us to triangulate data	Yes	Yes
Team reflexivity: We held weekly meetings to discuss discrepancies and refinements to coding processes	Yes	Yes

In addition, both approaches included processes to enhance methodological rigor [40, 41]. Credibility of results, a form of rigor, was most relevant when assessing tradeoffs between our rapid and traditional approaches [41]. We enhanced the credibility of results by having analysts with expertise in qualitative methods and the CFIR. To ensure participant responses were accurately captured in our summaries, we used two analysts per interview as a quality check and verified summaries with raw data (transcripts or audio recordings). Overall, the final summaries from both approaches were quite similar. See Table 5 for an additional description of the effectiveness and concordance of rigor between both approaches.

## Discussion

Our rapid deductive CFIR approach has much potential value, given the urgent need for nearly real-time results, to guide the implementation and dissemination of EBIs. The goal of this paper was to compare two qualitative approaches using deductively derived codes based on the CFIR: a traditional deductive CFIR approach using verbatim transcripts versus a rapid deductive CFIR approach using notes and audio recordings. Although we used the CFIR, this approach can be used with other frameworks. Our paper enhances the literature by describing exactly how rapid deductive CFIR analysis versus traditional deductive CFIR analysis leads to less resource use without compromising rigor.

Although our rapid deductive CFIR approach was beneficial for our evaluation team, researchers should review four considerations before using this method: (1) team expertise in CFIR and qualitative methods, (2) level of detail needed to meet project aims, (3) mode of data to analyze, and (4) advantages and disadvantages of using the CFIR.

First, the team’s expertise in the CFIR and qualitative methods should be considered before deciding to employ a rapid approach. Prior literature suggests that traditional qualitative analysis requires more intense training than rapid analysis [23, 28]. In-depth qualitative methods should indeed be conducted by a skilled research team. However, we argue that our rapid deductive CFIR approach may be more suited to researchers who already have a strong foundation in qualitative methods and the CFIR. Qualitative researchers familiar with the CFIR are more equipped to rapidly “code” qualitative data into CFIR constructs in real time than a novice. However, even for skilled researchers, we found that rapid analysis intensified effort and cognitive load during the initial coding phase, e.g., requiring a 3-h calendar block. Although a more experienced team may cost more in terms of salaries, the experienced team works more efficiently and likely saves money overall by reducing time spent training and overseeing project staff. For less experienced teams, we suggest linking CFIR constructs and brief definitions directly to interview questions within a notes template; this will help guide the researcher when summarizing the interview and/or listening to the audio recording. However, it is important to note that participant responses to questions will not always address the intended construct. Furthermore, while we identified a high level of fidelity between the primary analyst’s notes and the audio recordings, the secondary analyst may serve as an essential quality check for less experienced teams.

Second, researchers should consider what level of detail is needed for data analysis and the presentation of results in order to meet the project’s aims [28]. As articulated in prior research, rapid approaches using notes and audio recordings may provide a “big picture” view, yielding a lower level of detail than transcript-based approaches [28]. A project that requires a high level of detail and/or long quotations may therefore not be appropriate for our rapid approach. Our rapid CFIR approach provided less detail, but in so doing, may have allowed us to see both the overall patterns and the important details in our data more efficiently, i.e., seeing both the forest and the trees.

Third, the mode of data (transcripts or audio recordings) should be considered since it is not necessarily associated with a traditional or rapid approach. For example, audio recordings can be used for traditional analysis, i.e., many types of qualitative software allow minute-by-minute coding of audio recordings, and transcripts can be used for rapid analysis, i.e., summaries can be developed based on transcripts instead of audio recordings. For our rapid approach, we chose to use post-interview notes and audio recordings instead of transcripts to help streamline our deductive CFIR analysis process, i.e., it eliminated transcription costs and delays, and provided a point of comparison with other existing rapid approaches that use transcripts. However, if a team desires a more rapid approach while also maintaining access to the data in written form, including transcripts may be an option.

Fourth, there are advantages and disadvantages to consider when opting to use the CFIR (or another framework) regardless of the rapid or traditional qualitative analysis approach. Using the CFIR is helpful because it is a comprehensive determinant framework that includes constructs from 19 other models, including work by Greenhalgh et al. [42] that reviewed 500 published sources across 13 scientific disciplines. In effect, the CFIR helps researchers identify determinants that may be overlooked in a purely inductive approach. In addition, the use of the CFIR assists researchers with sharing and comparing results across studies, which advances implementation science. However, if researchers overly rely on the CFIR (or another framework), they may overlook constructs or miss important insights not included in the framework. To address this concern, we included questions in our interview guide beyond the scope of the CFIR, e.g., anticipated sustainment, and added codes, as needed, to capture inductively derived determinants and outcomes. Overall, even when using a more deductive approach, it is important for researchers to be open to inductive topics or domains that may arise in the data. Ultimately, researchers should consider their goals when deciding whether to adopt a deductive rapid approach (i.e., more confirmatory to compare with existing constructs or knowledge) versus an inductive approach (i.e., more exploratory to generate new constructs or knowledge).

It is important to note that our rapid deductive CFIR approach was still time intensive; it took 409.5 h to complete the analysis, including the rating process, for cohort B. However, because the analysts completed interview notes and coding in the matrix immediately after each interview, we were able to share preliminary results during regularly scheduled meetings with our operational partners on an ongoing basis. Regardless, some researchers may need additional ways to streamline our rapid CFIR analysis process. As long as a team considers both strengths and limitations, the following strategies may provide ways to streamline our rapid CFIR approach:

- The team could eliminate the second analyst entirely or only use a second analyst on a subset of interviews, e.g., on the first 10 interviews or a random sample.
- The team could include only the CFIR constructs expected to be most relevant to the research question in the matrix.
- The team could seek to obtain project artifacts, e.g., meeting minutes, to analyze in the place of interviews.
- The team could omit the rating process following coding.

Although rapid approaches are becoming more alluring to many implementation science researchers, they should not be considered a quick and easy replacement for traditional approaches or a substitute for having a skilled research team. Teams must carefully consider the best approach for their project while also exploring how to maintain scientific rigor. Qualitative expert oversight and/or training, analyst familiarity with the framework, review by a secondary analyst, and interview data quality are some important aspects of methodological rigor.

### Limitations

Several limitations should be noted. First, both analysts on this project were intermediate to expert CFIR users. Our approach may be more difficult for new CFIR users, i.e., it may be difficult to translate interview notes into “coded” data in the matrix or to “code” while listening to an audio recording, unless the researchers are very familiar with the constructs. Second, the same analysts were involved in analyzing both cohorts. It is possible the analysts were more familiar with the broader findings from the study based on the traditional analysis of cohort A, which may have allowed them to progress more quickly in the rapid analysis of cohort B. However, using the same analysts improves comparability of coding between the two different cohorts of data and streamlined the process because additional analysts did not need to be trained in using the CFIR. Future research is needed to assess the extent and the conditions under which our approach works for other CFIR users. Third, we focused on differences in time and transcription costs rather than specifically testing the effectiveness or rigor of our rapid versus traditional approach, which has been discussed in prior literature [23, 28]. While the rigor of the results was the same with both approaches, future researchers should likewise assess the rigor of this deductive rapid approach within their circumstances.

## Conclusions

Our deductive rapid approach using the CFIR, involving notes and audio recordings, is an effective and rigorous approach for analyzing qualitative data that resulted in substantial reductions in time and transcription costs. We intend to use this approach for similar studies in the future. Overall, a deductive rapid approach using the CFIR (or another framework) is especially beneficial when (1) the research team has strong qualitative methods and skills using the framework, (2) the research timeline is relatively short or real-time feedback is needed, (3) the budget  does not support transcription, and (4) the research team wants to compare results across studies.

## Supplementary Information

**Additional file 1.** Interview Guide.

**Additional file 2.** Unabridged MS Word CFIR Facility Memo Template.

**Additional file 3.** Unabridged MS Excel CFIR Construct by Facility Matrix Template.

## Data Availability

The datasets generated and/or analyzed during the current evaluation are not available due to participant privacy but may be available from the corresponding author on reasonable request.

## Abbreviations

CFIR: Consolidated Framework for Implementation Research
DoE: Diffusion of Excellence
EBI: Evidence-Based Innovation
VHA: Veterans Health Administration
