# Supplementary material for: Rapid versus traditional qualitative analysis using the Consolidated Framework for Implementation Research (CFIR)
Source: Implement Sci. 2021 Jul 2;16:67. doi: 10.1186/s13012-021-01111-5 (PMC8252308; doi:10.1186/s13012-021-01111-5)
Supplement: Supplementary file 1 — Additional file 1. Interview Guide. [file 13012_2021_1111_MOESM1_ESM.docx]

| **Additional File 1. CFIR Informed Interview Guide** | | |
| --- | --- | --- |
| **Semi-Structured Interview Guide** | | |
| **CFIR Construct(s)** | **Question** | **Probe(s)** |
| Formally Appointed Internal Implementation Leader (FIL) | What is your role at your site (outside of implementation)?  How long have you worked for the VA? |  |
| External Policies and Incentives (EPI)  Patient Needs & Resources (PNR)  Staff Needs & Resources (SNR)  Tension for Change (TFC) | Why did your site choose to implement the practice? | What level of need did you/others see for the practice?  If participant does not elaborate: Why did you see that level of need? |
| Leadership Engagement (LE)  Formally Appointed Internal Implementation Leader (FIL)  Design Quality & Packaging (DQP) | Who was involved in reviewing practices and developing the bid? | How did you learn that your site had bid on the practice?  When/how did you learn about the details of the practice?  How well did the video and presentation describe the components and aim of the practice?  Did you understand what was needed (e.g., infrastructure, staff, resources) to implement the practice? |
| Implementation Readiness:  Compatibility (COMP)  Key Stakeholders: Intervention Team (INT KS)  Available Resources (AR) | Was your facility ready to implement the practice based on necessary infrastructure? Staff? Resources? | If participant does not elaborate:  Tell me more about infrastructure  Tell me more about staffing  Tell me more about resources |
| Compatibility (COMP)  Relative Advantage (RA) | Did the practice fit with existing work processes at your site? | If participant does not elaborate: Why or why not?  Did you have anything similar to the practice already in place?  If participant does not elaborate: Tell me more about that?  What were advantages and disadvantages to the practice compared to the old process? |
| Leadership Engagement (LE)  Formally Appointed Internal Implementation Leader (FIL) | How did you become the Implementing Facility Fellow?  How well suited were you to lead implementation?  Had you ever implemented a new process/program? | If participant does not elaborate: If yes, can you tell me more about that experience? |
| External Change Agent (ECA)  Access to Knowledge & Information (AKI) | Which activities or kinds of support were most/least helpful from the DoE Team Lead (Atlas) and QUERI Hub Coach in helping you implement the practice? |  |
| Key Stakeholders: Implementation Team (IMP KS) | Who was on the implementation team at your site and what was their role? | How did you engage them? |
| Leadership Engagement (LE) | What was the role of leadership? | How would you describe their level of support during implementation? |
| Open CFIR Constructs | You have already mentioned some challenges…  What made implementation more difficult?  What made implementation easier? | What strategies were used to overcome challenges? |
| Adapting (ADPT) | What kinds of adaptations were made to the practice? | Why were these adaptations made? |
| Now I want to talk about how you think about success in terms of the practice implementation. | | |
| Implementation Success (IMP SUC) | Was implementation completed by the 6-month facilitation deadline?    Overall, from a scale of 1 to 10, where 1 is unsuccessful, and 10 is successful, how successful was your site *implementing* the practice? | If participant does not elaborate: Why or why not?  Were you aware that implementation was intended to be completed within 6 months?  How long should the implementation timeline be for this practice?  **Note: Ask the question exactly as written.**  Why did you choose that number?  What would it take to increase this number? |
| Implementation Success (IMP SUC) | If you could go back, what would you do differently during implementation? | What did you learn that would be relevant to another site implementing the practice? |
| Intervention Success (INT SUC) | How will your site define and measure the practice effectiveness? |  |
| Sustainability (SUSTAIN) | How likely is it that the practice will be sustained at your site? | If participant does not elaborate: Why would you say that? |
| Design Quality & Packaging (DQP) | Thinking back to the beginning of facilitated implementation, how ready was the practice to be implemented in another facility in terms of maturity and design quality?  Following facilitated implementation, how ready is the practice now? | If the participant does not elaborate: please tell me more about that  Determine when the practice had:   - Implementation toolkit and materials - National level approvals   - OIT   - Union - National Stakeholders/Endorsements - Supporting data |
| Diffusion (DIFFUSE) | Should this practice be diffused nationally? | If the participant does not elaborate: Why or why not? |
| Interview Conclusion | Based on your experience, overall, from a scale of 1 to 10, where 1 is unsuccessful, and 10 is successful, how successful is the  DoE as model of diffusion?  Is there anything else that you would like to add to our conversation today? | Why did you choose that number?  What suggestions do you have for the DOE/ future Shark Tanks? |
