# Supplementary material for: Rapid versus traditional qualitative analysis using the Consolidated Framework for Implementation Research (CFIR)
Source: Implement Sci. 2021 Jul 2;16:67. doi: 10.1186/s13012-021-01111-5 (PMC8252308; doi:10.1186/s13012-021-01111-5)
Supplement: Supplementary file 2 — Additional file 2. Unabridged MS Word CFIR Facility Memo Template. [file 13012_2021_1111_MOESM2_ESM.docx]

**Additional File 2. CFIR Facility Memo Template**

**Analysts:**

**Facility:**

**Interview Participants:**

**High-Level Facility Summary:**

[Provide high-level facility summary]

**CFIR Constructs:**

Note: Enhance the memo by including additional topics/domains identified during coding.

**I. INNOVATION CHARACTERISTICS**

**A Innovation Source**

*RATING: OVERALL __ (ANALYST ONE __, ANALYST TWO __)*

*SUMMARY:*

[Provide summary of coded data]

*RATIONALE:*

[Provide rationale for rating]

*DATA:*

[Copy coded data from software]

**B Evidence Strength & Qualit**y

*RATING: OVERALL ___ (ANALYST ONE ___, ANALYST TWO ___)*

*SUMMARY:*

*RATIONALE:*

*DATA:*

**C Relative Advantage**

*RATING: OVERALL ___ (ANALYST ONE ___, ANALYST TWO ___)*

*SUMMARY:*

*RATIONALE:*

*DATA:*

**D Adaptability**

*RATING: OVERALL ___ (ANALYST ONE ___, ANALYST TWO ___)*

*SUMMARY:*

*RATIONALE:*

*DATA:*

**E Trialability**

*RATING: OVERALL ___ (ANALYST ONE ___, ANALYST TWO ___)*

*SUMMARY:*

*RATIONALE:*

*DATA:*

**F Complexity** (Reverse rated, low complexity understood as positive)

*RATING: OVERALL ___ (ANALYST ONE ___, ANALYST TWO ___)*

*SUMMARY:*

*RATIONALE:*

*DATA:*

**G Design Quality & Packaging**

*RATING: OVERALL ___ (ANALYST ONE ___, ANALYST TWO ___)*

*SUMMARY:*

*RATIONALE:*

*DATA:*

**H Cost**

*RATING: OVERALL ___ (ANALYST ONE ___, ANALYST TWO ___)*

*SUMMARY:*

*RATIONALE:*

*DATA:*

**II. OUTER SETTING**

**A Needs & Resources of Those Served by the Organization**

*RATING: OVERALL ___ (ANALYST ONE ___, ANALYST TWO ___)*

*SUMMARY:*

*RATIONALE:*

*DATA:*

**B Cosmopolitanism**

*RATING: OVERALL ___ (ANALYST ONE ___, ANALYST TWO ___)*

*SUMMARY:*

*RATIONALE:*

*DATA:*

**C Peer Pressure**

*RATING: OVERALL ___ (ANALYST ONE ___, ANALYST TWO ___)*

*SUMMARY:*

*RATIONALE:*

*DATA:*

**D External Policy & Incentives**

*RATING: OVERALL ___ (ANALYST ONE ___, ANALYST TWO ___)*

*SUMMARY:*

*RATIONALE:*

*DATA:*

**III. INNER SETTING**

**A Structural Characteristics**

*RATING: OVERALL ___ (ANALYST ONE ___, ANALYST TWO ___)*

*SUMMARY:*

*RATIONALE:*

*DATA:*

**B Networks & Communications**

*RATING: OVERALL ___ (ANALYST ONE ___, ANALYST TWO ___)*

*SUMMARY:*

*RATIONALE:*

*DATA:*

**C Culture**

*RATING: OVERALL ___ (ANALYST ONE ___, ANALYST TWO ___)*

*SUMMARY:*

*RATIONALE:*

*DATA:*

**D Implementation Climate**

***1 Tension for Change***

*RATING: OVERALL ___ (ANALYST ONE ___, ANALYST TWO ___)*

*SUMMARY:*

*RATIONALE:*

*DATA:*

***2 Compatibility***

*RATING: OVERALL ___ (ANALYST ONE ___, ANALYST TWO ___)*

*SUMMARY:*

*RATIONALE:*

*DATA:*

***3 Relative Priority***

*RATING: OVERALL ___ (ANALYST ONE ___, ANALYST TWO ___)*

*SUMMARY:*

*RATIONALE:*

*DATA:*

***4 Organizational Incentives & Rewards***

*RATING: OVERALL ___ (ANALYST ONE ___, ANALYST TWO ___)*

*SUMMARY:*

*RATIONALE:*

*DATA:*

***5 Goals & Feedback***

*RATING: OVERALL ___ (ANALYST ONE ___, ANALYST TWO ___)*

*SUMMARY:*

*RATIONALE:*

*DATA:*

***6 Learning Climate***

*RATING: OVERALL ___ (ANALYST ONE ___, ANALYST TWO ___)*

*SUMMARY:*

*RATIONALE:*

*DATA:*

**E Readiness for Implementation**

***1 Leadership Engagement***

*RATING: OVERALL ___ (ANALYST ONE ___, ANALYST TWO ___)*

*SUMMARY:*

*RATIONALE:*

*DATA:*

***2 Available Resources***

*RATING: OVERALL ___ (ANALYST ONE ___, ANALYST TWO ___)*

*SUMMARY:*

*RATIONALE:*

*DATA:*

***3 Access to Knowledge & Information***

*RATING: OVERALL ___ (ANALYST ONE ___, ANALYST TWO ___)*

*SUMMARY:*

*RATIONALE:*

*DATA:*

**IV. CHARACTERISTICS OF INDIVIDUALS**

**A Knowledge & Beliefs about the Innovation**

*RATING: OVERALL ___ (ANALYST ONE ___, ANALYST TWO ___)*

*SUMMARY:*

*RATIONALE:*

*DATA:*

**B Self-Efficacy**

*RATING: OVERALL ___ (ANALYST ONE ___, ANALYST TWO ___)*

*SUMMARY:*

*RATIONALE:*

*DATA:*

**C Individual Stage of Change**

*RATING: OVERALL ___ (ANALYST ONE ___, ANALYST TWO ___)*

*SUMMARY:*

*RATIONALE:*

*DATA:*

**Individual Identification with Organization**

*RATING: OVERALL ___ (ANALYST ONE ___, ANALYST TWO ___)*

*SUMMARY:*

*RATIONALE:*

*DATA:*

**E Other Personal Attributes**

*RATING: OVERALL ___ (ANALYST ONE ___, ANALYST TWO ___)*

*SUMMARY:*

*RATIONALE:*

*DATA:*

**V. PROCESS**

**A Planning**

*RATING: OVERALL ___ (ANALYST ONE ___, ANALYST TWO ___)*

*SUMMARY:*

*RATIONALE:*

*DATA:*

**B Engaging**

*RATING: OVERALL ___ (ANALYST ONE ___, ANALYST TWO ___)*

*SUMMARY:*

*RATIONALE:*

*DATA:*

***1 Opinion Leaders***

*RATING: OVERALL ___ (ANALYST ONE ___, ANALYST TWO ___)*

*SUMMARY:*

*RATIONALE:*

*DATA:*

***2 Formally Appointed Internal Implementation Leaders***

*RATING: OVERALL ___ (ANALYST ONE ___, ANALYST TWO ___)*

*SUMMARY:*

*RATIONALE:*

*DATA:*

***3 Champions***

*RATING: OVERALL ___ (ANALYST ONE ___, ANALYST TWO ___)*

*SUMMARY:*

*RATIONALE:*

*DATA:*

***4 External Change Agents***

*RATING: OVERALL ___ (ANALYST ONE ___, ANALYST TWO ___)*

*SUMMARY:*

*RATIONALE:*

*DATA:*

***5 Key Stakeholders***

*RATING: OVERALL ___ (ANALYST ONE ___, ANALYST TWO ___)*

*SUMMARY:*

*RATIONALE:*

*DATA:*

***6 Innovation Participants***

*RATING: OVERALL ___ (ANALYST ONE ___, ANALYST TWO ___)*

*SUMMARY:*

*RATIONALE:*

*DATA:*

**C Executing**

*RATING: OVERALL ___ (ANALYST ONE ___, ANALYST TWO ___)*

*SUMMARY:*

*RATIONALE:*

*DATA:*

**D Reflecting & Evaluating**

*RATING: OVERALL ___ (ANALYST ONE ___, ANALYST TWO ___)*

*SUMMARY:*

*RATIONALE:*

*DATA:*
